# Subacute Ruminal Acidosis and Evaluation of Blood Gas Analysis in Dairy Cow

**DOI:** 10.4061/2010/392371

**Published:** 2010-09-29

**Authors:** Matteo Gianesella, Massimo Morgante, Chiara Cannizzo, Annalisa Stefani, Paolo Dalvit, Vanessa Messina, Elisabetta Giudice

**Affiliations:** ^1^Department of Veterinary Clinical Sciences, University of Padua, Viale dell'Università 16, Legnaro, 35020, (Padua), Italy; ^2^Experimental Zooprophyilactic Institute of Venices, Viale dell'Università 10, Legnaro, 35020, (Padua), Italy; ^3^Department of Experimental Sciences and Applied Biotechnologies, Faculty of Veterinary Medicine, University of Messina, Polo Universitario SS Annunziata, 98168, Messina, Italy; ^4^Department of Veterinary Public Health, Faculty of Veterinary Medicine, University of Messina, Polo Universitario SS Annunziata, 98168, Messina, Italy

## Abstract

Subacute Ruminal Acidosis (SARA) corresponds to an imbalance between lactate-producing bacteria and lactate-using bacteria, which results in a change in ruminal pH associated with a prevalent consumption of rapidly fermentable carbohydrates. In our study, 216 primiparus and multiparus dairy cows were selected from 20 Italian intensive dairy herds and were divided into three groups based on the risk of SARA. All the dairy cows had high average milk production. After blood sampling, a complete blood gas analysis was performed. One-way ANOVA was performed to compare the three groups. O_2_ Cont, PCO_2_, blood pH, O_2_Hb, urinary pH, and rumen pH were significantly lower in cows with rumen pH < 5.5. These results indicate that blood gas analysis is a valuable tool to diagnose acidosis in dairy cows because it provides good assessment of acidosis while being less invasive than rumen pH analysis.

## 1. Introduction

Subacute Ruminal Acidosis (SARA) is a complex pathology frequently encountered in high-production dairy cows. To achieve incremented feed conversion, cattle, sheep, and goats are fed a high-energy diet that eventually causes various digestive disorders [1]. The rumen mucosa plays a vital role in whole energy balance trough transport and metabolism of rumen-derived volatile fatty acids [1].

 The susceptibility of dairy cows to SARA seems to be higher for cows in early lactation, probably due to the instability of the bacterial population [2]. In fact, the onset of this pathology corresponds to an imbalance between lactate-producing bacteria and lactate-using bacteria [2, 3]. This disequilibrium is due to a change in the rumen pH, related to prevalent consumption of rapidly fermentable carbohydrates [2]. Usually the rumen pH threshold below which acidosis occurs is 5.5 [4]. Despite a similarity in rumen pH, SARA should not be confused with the chronic acidosis and with the lactic acidosis that are typical of beef cattle as an acute pathological process caused by indigestion of cereals [4]. The digestion process is also inhibited by an excessive distension of the rumen's wall, which leads to a reduction in motility and an accumulation of volatile fatty acids (VFA) inside the organ, which further alters microbial status with an excessive production of propionate, a fatty acid inhibiting the digestion process [4]. The result is a lowering of the rumen pH as well as a shifting of the rumen fluid's buffer capacity to an area around pH 5 [5]. This pathological complex can affect behavioural patterns and cause severe production losses [1, 3, 4, 6]. Reduced milk production, ruminitis, and parakeratosis often occur as a consequence of absorption of ruminal bacteria in the circulation [4, 7]. Herbivores, including bovines, absorb organic acids, lactate, and carbohydrates in the rumen, and the pH of body fluids depends on the degree of compensation of bicarbonate buffer [8]. The effect of a change in the dietary cation-anion difference (DCAD) is an increase in blood pH and HCO^3−^ concentration and an excess of bases in the blood. Because blood is the main vehicle of toxins and other products of fermentation, blood gas analysis can be a useful means to detect the early onset of the pathology. On the basis of such considerations, the aim of this paper was to examine the modifications of blood gas analysis in cattle from intensive dairy farms, some of which were in a condition of high risk for SARA.

## 2. Materials and Methods

### 2.1. Farm and Nutrition

Twenty Italian intensive dairy herds were selected from different areas throughout northern Italy, some of which were considered potentially at high risk of SARA. All the herds had a high average milk production (about 10000 Kg per year). The dairy cows were housed in free stalls and, in the early part of their lactations, had use of total mixed ration (TMR) and adoption of “steaming up” in the final part of the dry period as standard farming practice.

### 2.2. Animals

In total, 216 cows were randomly selected from all the farms and were divided into three groups of 72 animals each. All the animals were Holstein breed and were in the first 60 days of lactation. At a general physical examination, all the subjects were clinically healthy. Table 1 shows the chemical composition analysis of the diet administered during the study. The Body Condition Score (BCS) average values were 3.03 ± 0.07, in a 1 to 5 scale, according with the procedure of Edmonson et al. [9].

### 2.3. Ruminal Samples

All the subjects of our study were subjected to a rumenocentesis, using a 13 G 105-mm needle (Intranule PP, Vygon, France). This procedure was chosen because it is the most commonly used technique and provides the most accurate results [10–12]. The time of sampling was between 4 and 6 hours post TRM distribution, as recommended by Morgante et al. [12]. An area in the left flank of 20 × 20 cm, 20 cm caudal to the last costae, and on the level of the top of the stifle joint was prepared with an aseptic technique by disinfection with ethanol and iodine. The farmer was instructed to restrain the dairy cows by means of a tail grip, and the needle was introduced into the rumen by a veterinary surgeon. The pH of the rumen fluid was immediately determined by means of a portable pHmeter (Piccolo, Hanna Instruments). All the herds were classified into three different groups in relation to the acidosis risk, depending on the rumen pH. Group A was composed by subjects with a pH > 5.8, group B was composed by subjects with a pH ≤ 5.5 ≤ 5.8, and group C by subjects with a pH < 5.5.

### 2.4. Blood Samples

Blood samples were collected by jugular venipuncture using a blood sampling kit for blood gas analysis (3 ml ventilated syringes with 23 G × 1 in needle, containing freeze-dried lithium heparin, Nova Biomedical Corp, USA). All the samples were immediately analysed in a calibrated blood gas analyser (“Stat Profile pHOx” blood gas analyzer, Nova Biomedical Corp., USA), set at the body temperature of the cow, and the following parameters were determined: haemoglobin (Hgb) by a combination of conductivity and photometric measurements, hematocrit (HCT) by blood electrical-resistance measurement, oxygen content (O_2_ Ct), partial pressure of Oxygen (PO_2_) and partial pressure of carbon dioxide (PCO_2_) by the Severinghaus method, and hydrogen ion activity in blood (pH) by direct ISE. The following parameters were obtained through calculation: base excess (BE-b), base excess in extracellular fluid (BE-ecf), standard bicarbonate concentration (SBC), bicarbonate level (HCO_3_^−^), total carbon dioxide (TCO_2_), and oxyhemoglobin (O_2_ HB). Measurements were carried out as recommended by the National Committee of Blood Laboratory Standards. (*Considerations in the Simultaneous Measurement of Blood Gases, Electrolytes and Related Analytes in Whole Blood; Proposed Guidelines.*) For each test, the analyser operating temperature was set according to the bovine rectal temperature recorded during sampling.

### 2.5. Urinary Sample

Urine samples were collected by catheterism early in the morning (8.00 AM) and stored in glass tubes. Immediately after collection, urinary pH was measured by means of a portable pHmeter (Piccolo Hanna Instruments, Leighton Buzzard, Bedfordshire, UK).

### 2.6. Statistical Analysis

One-way Analysis of Variance (ANOVA) was applied to compare all groups. The Bonferroni's test was applied for post hoc comparison. A *P value *< .05 was considered statistically significant. All data were analyzed using Statistica 7 software (Statsoft Inc.).

## 3. Results and Discussion

Table 2 shows the mean values of the considered parameters expressed in their units of measurement, with the relative standard deviation and statistical significance, recorded during our experiment for the three groups. ANOVA revealed a significant difference among the groups, with pairwise post hoc comparions showing a significant difference between group C and the other groups, for the following parameters: O_2_ Cont (*P* < .001, *F*_(2;215)_ = 5.90) PCO_2_ (*P* < .05, *F*_(2;215)_ = 3.24), blood pH (*P* < .005, *F*_(2;215)_ = 5.50), PO_2_ (*P* < .001, *F*_(2;215)_ = 19.55), O_2_Hb (*P* < .001, *F*_(2;215)_ = 5.93), urinary pH (*P* < .0001, *F*_(2;215)_ = 24.92), and rumen pH (*P* < .001, *F*_(2;215)_ = 5.85). Post hoc comparisons revealed no significant differences between groups A and B. 

**Figure 1:**
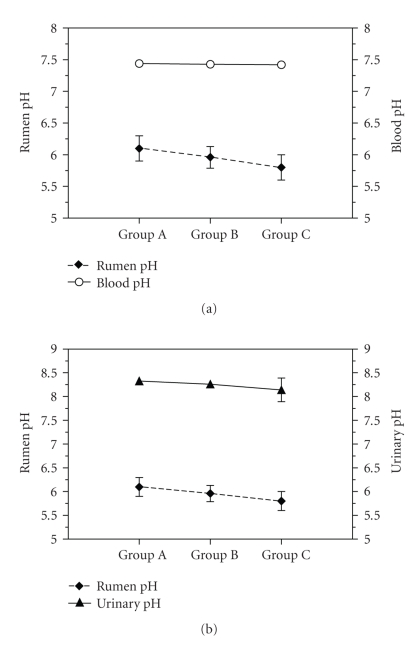
Graphical representation of the trends of rumen pH and blood pH (a) and rumen pH and urinary pH (b), in three experimental groups (group A, B and C), each composed of 72 dairy cows, which differed for Ruminal pH.

Figure 1 shows the graphical representation of the values of rumen pH, blood pH, and urinary pH, recorded during our experimental conditions. 

There is a tight relationship between the rumen pH and blood pH [13]. In our study the blood pH had a lower value in group C, as acidosis is characterized by a blood pH lower than normal [8]. In newborn calves, a reduced absorption of colostrum, is due to hypercapnia which is a clinical sign of acidosis [14, 15]. In adults and, particularly, in ruminants, blood pH depends on the relative concentrations of bases, acids, and buffers in solution [8]. Low blood concentration of ammonia does not affect blood pH, and the only buffer is bicarbonate [8]. A base excess is normally present in blood, but a load of acids such as that of acidosis can decrease the base excess and, consequently, can overcome the buffering capacity of bicarbonate [13]. A significant decrease in groups B and C was recorded for urinary pH, although the urinary pH values obtained were not pathological. Most excreted urine H^+^are associated with buffers or ammonia, in addition to free H^+^ excreted in the urine [16]. As reported by other authors, a high positive DCAD can lead to an imbalanced acid base status, resulting in a change in urinary pH [16, 17]. However, the DCAD value of the diet administered in our study was not sufficient to alterate the urinary pH, so the values found were within the normality range. It is very interesting that in our study blood pH was inversely related to rumen pH. Both O_2_Hb and O_2_ content are parameters strictly related to blood oxygenation, and the lowest values were recorded in group C. The same trend was seen for PO_2_ which, compared to the previous two parameters, is the most commonly used measure. Thus, the decrease of the values of oxygenation can be attributed to an increase in the anaerobic metabolism and consequently an increase of oxygen consumption [18]. In contrast, the partial pressure of carbon dioxide (PCO_2_) was higher in group C, which confirms the diagnosis of SARA for the dairy cows from group C.

## 4. Conclusions

These results indicate that blood gas analysis is a valuable tool to diagnose acidosis in dairy cows because it provides good assessment of acidosis while being less invasive than rumen pH analysis. Moreover, blood gas analysis can help us to differentiate respiratory acidosis from metabolic acidosis, especially in a subacute form such as SARA. Further investigations should be conducted to evaluate the differences between SARA and other forms of metabolic diseases that affect dairy herds the most.

## Figures and Tables

**Table 1:** Mean values of the chemical composition of the diet administered to 216 cows randomly selected from 20 dairy herds.

Parameters	Mean ± SD
Crude protein (g/Kg)	15.21 ± 1.06
Ethereal extract (%)	4.52 ± 0.43
Ash (%)	7.48 ± 0.85
NDF (%)	35.30 ± 1.83
NSC (%)	37.46 ± 2.47
Digestible dry matter (%ss)	65.48 ± 1.85
ADF (acid detergent fiber)	20.58 ± 1.66
Starch (g/Kg)	22.87 ± 1.97
Calcium (mg/Kg)	1.00 ± 0.05
Phosphorus (mg/Kg)	0.45 ± 0.01
Magnesium (mg/Kg)	0.33 ± 0.02
Sodium (mg/Kg)	0.58 ± 0.05
Potassium (mg/Kg)	1.45 ± 0.10
Chlorine (mg/Kg)	0.32 ± 0.09
Anions/cations (meq/100 gr)	41.78 ± 2.09
NDF/NSC	0.94 ± 0.10
NSC/NDF	1.06 ± 0.11
NDF/proteins	2.33 ± 0.22
Starch/proteins	1.50 ± 0.14

**Table 2:** Mean values (±SD) of haematological parameters in dairy cows of Groups A, B, and C, each composed of 72 dairy cows. Significant differences between group C and the other two groups are indicated by * (vs group A, *P* < .05) and ^●^ (vs group B, *P* < .001).

	Parameters	Experimental Groups		
		Group A	Group B	Group C
Measured parameters	Rumen pH	6.10 ± 0.38	5.96 ± 0.30	5.80 ± 0.35^∗●^
	Urinary pH	8.33 ± 0.07	8.26 ± 0.05*	8.14 ± 0.25*
	Hgb (g/dL)	10.06 ± 0.10	10.21 ± 0.09	10.02 ± 0.10
	HCT (%)	31.71 ± 0.36	32.44 ± 0.35	31.74 ± 0.39
	Blood pH	7.44 ± 0.00	7.43 ± 0.00	7.42 ± 0.00*
	PO_2_ (mmHg)	38.03 ± 3.62	37.06 ± 4.70	34.03 ± 3.50^∗●^
	PCO_2_ (mmHg)	44.10 ± 0.47	44.69 ± 0.37	45.60 ± 0.40*

Calculated parameters	BE B (mmol/L)	6.01 ± 0.23	6.19 ± 0.29	5.98 ± 0.23
	BE ECF (mmol/L)	5.91 ± 0.27	6.06 ± 0.33	5.85 ± 0.26
	SBC (mmol/L)	29.08 ± 0.17	29.22 ± 0.22	28.90 ± 0.17
	TCO_2_ (mmol/L)	31.62 ± 0.27	31.85 ± 0.32	31.82 ± 0.25
	O_2_HB	67.72 ± 1.05	68.55 ± 0.94	63.79 ± 1.12^∗●^
	O_2_ Cont (ml/dL)	9.94 ± 0.19	10.32 ± 0.18	9.37 ± 0.21^●^
	HCO^3−^ (mmol/L)	30.25 ± 0.25	30.48 ± 0.21	30.41 ± 0.24
